# Cross-Serotype Immunity Induced by Immunization with a Conserved Rhinovirus Capsid Protein

**DOI:** 10.1371/journal.ppat.1003669

**Published:** 2013-09-26

**Authors:** Nicholas Glanville, Gary R. Mclean, Bruno Guy, Valerie Lecouturier, Catherine Berry, Yves Girerd, Christophe Gregoire, Ross P. Walton, Rebecca M. Pearson, Tatiana Kebadze, Nicolas Burdin, Nathan W. Bartlett, Jeffrey W. Almond, Sebastian L. Johnston

**Affiliations:** 1 Airways Disease Infection Section, National Heart and Lung Institute, Medical Research Council and Asthma United Kingdom Centre in Allergic Mechanisms of Asthma, Centre for Respiratory Infections, Imperial College London, London, United Kingdom; 2 Discovery Department, Sanofi Pasteur, Campus Merieux, Marcy l'Etoile, France; Nationwide children's Hospital and Ohio State University, United States of America

## Abstract

Human rhinovirus (RV) infections are the principle cause of common colds and precipitate asthma and COPD exacerbations. There is currently no RV vaccine, largely due to the existence of ∼150 strains. We aimed to define highly conserved areas of the RV proteome and test their usefulness as candidate antigens for a broadly cross-reactive vaccine, using a mouse infection model. Regions of the VP0 (VP4+VP2) capsid protein were identified as having high homology across RVs. Immunization with a recombinant VP0 combined with a Th1 promoting adjuvant induced systemic, antigen specific, cross-serotype, cellular and humoral immune responses. Similar cross-reactive responses were observed in the lungs of immunized mice after infection with heterologous RV strains. Immunization enhanced the generation of heterosubtypic neutralizing antibodies and lung memory T cells, and caused more rapid virus clearance. Conserved domains of the RV capsid therefore induce cross-reactive immune responses and represent candidates for a subunit RV vaccine.

## Author Summary

Human rhinovirus infections cause the majority of common colds as well as asthma and chronic obstructive pulmonary disease (COPD) exacerbations. The disease burden attributable to rhinoviruses is therefore huge. Despite this and the fact that human rhinoviruses were discovered over 50 years ago, there are currently no specific antiviral therapies or vaccine available. The lack of a rhinovirus vaccine can at least in part be attributed to the fact that rhinoviruses like other pathogens have high variability in surface antibody binding regions, resulting in >100 serotypically distinct strains. We have defined areas of the rhinovirus polyprotein which are highly conserved across strains and which may therefore induce cross-reactive immune responses capable of providing broader protection. Using a mouse model, we show that immunization with a recombinant rhinovirus capsid protein induces cross-reactive cellular and humoral immune responses. After subsequent infection, immunization enhances both neutralising antibody and lung effector and memory T cell responses, expediting virus clearance. Importantly these effects were evident upon challenge with multiple heterologous rhinovirus serotypes, indicating that immunization with conserved rhinovirus capsid proteins may represent a viable strategy for producing a broadly cross-reactive vaccine.

## Introduction

Human rhinovirus (RV) infections are the most frequent cause of the common cold [1] and are highly associated with exacerbations of asthma and COPD [2], [3], [4]. Despite the great disease burden and healthcare costs therefore attributable to RV infections, there is currently neither a vaccine nor specific anti-viral therapy available.

The requirements for immunity to RV are poorly understood. Experimental and natural infections induce antibodies which provide some protection against re-infection with the same RV serotype [5], [6], [7]. Intranasal and intramuscular inactivated virus vaccinations similarly induce neutralizing antibodies and provide protection against disease induced with the same RV serotype [8], [9]. There are however greater than 100 serotypes of RV [10], divided into major and minor groups based on receptor usage and A and B groups based on antiviral sensitivity and nucleotide sequence [11], [12], and a further ∼50–60 RV species more recently defined as group C RVs based on sequence data alone [13], [14]. Serological variability amongst RVs therefore means that vaccines designed to generate neutralizing antibodies are unlikely to provide sufficiently broad protection to prevent the frequent infections which occur throughout life.

Alternative vaccination strategies based on inducing T cell responses to conserved antigens have been explored for a number of pathogens, including respiratory viruses [15], [16]. An advantage of this approach lies in the ability of T cells to recognize internal virus proteins which are typically more highly conserved than surface exposed regions containing neutralizing antibody epitopes. T cells are therefore potentially cross-reactive against different virus strains, as has been shown with influenza viruses [17], [18], for which surface antigenic variability is also an obstacle to effective vaccine design.

For RVs, naturally occurring memory T cells can be cross-serotype responsive [19], [20] and immunization with RV peptides has been suggested to be capable of inducing cross-serotype reactive T cells in mice [21]. Most of the naturally occurring RV-specific memory T cells characterized to date have shown a Th1/Tc1 bias [19], [20]. *In vitro* responses to RV by mixed PBMCs have been associated with virus shedding or cold symptoms after subsequent infection [22] but there is no evidence that naturally occurring RV-specific memory T cells specifically provide benefit in terms of virus control or disease symptoms *in vivo*. Here we show that a vaccine composition which elicits a Th1/Tc1 biased T cell response to conserved RV antigens could have efficacy.

We took a bioinformatic approach to identify regions of the RV polyprotein which are conserved across A and B group and major and minor receptor binding group viruses, and which might be used as immunogens in a cross-reactive vaccine. As in similar analyses by others [11], we show that areas of the capsid VP0 protein are highly conserved amongst RVs. Immunization with VP0 protein from major group RV16 combined with Th1 promoting adjuvants induced antigen-specific, type I orientated T cell responses in the airways, enhanced neutralizing antibody responses to infection and caused a more rapid decrease in lung virus load in mice. Importantly, these effects were seen in mice infected with heterologous RV strains, indicating that capsid protein immunization could provide broadly cross-reactive immunity against RVs.

## Results

### The VP0 protein is highly conserved amongst RVs

Using published amino acid sequences we defined areas of the RV polyprotein which are conserved across A and B species RVs. The methodology for determining amino acid sequence conservation amongst RVs is described in materials and methods. We did not find well conserved sequences covering both A and B species RVs, but within each species three regions were identified as highly conserved in agreement with similar sequence comparisons carried out previously [11] and therefore represented candidate antigens. These were amino acids 1–191 and 243–297 in the N-terminus of the polyprotein, and the C-terminal domain of the RNA polymerase (Fig. S1a). The two N-terminus regions lie within the VP4 and VP2 capsid proteins, of which VP0 is the natural precursor. Because VP0 contains both very highly conserved internal (VP4) and surface exposed regions with neutralizing epitopes (VP2), VP0 was chosen as the antigen for further studies. Sequences from RV16, a major group A species RV were used to allow study of cross-reactivity to minor group RV strains which can infect wild type mice [23]. Figure S1 shows detailed analysis of the high sequence conservation within VP0 (Fig. S1b), the amino acid sequence of the RV16 VP0 immunogen (Fig. S1c) and comparison of RV16 VP0 with VP0 sequences of minor group RVs 1B, 29 and 14 used subsequently (Fig. S1d).

### Immunization induces a VP0 specific, cross-serotype immune response

**Figure 1:**
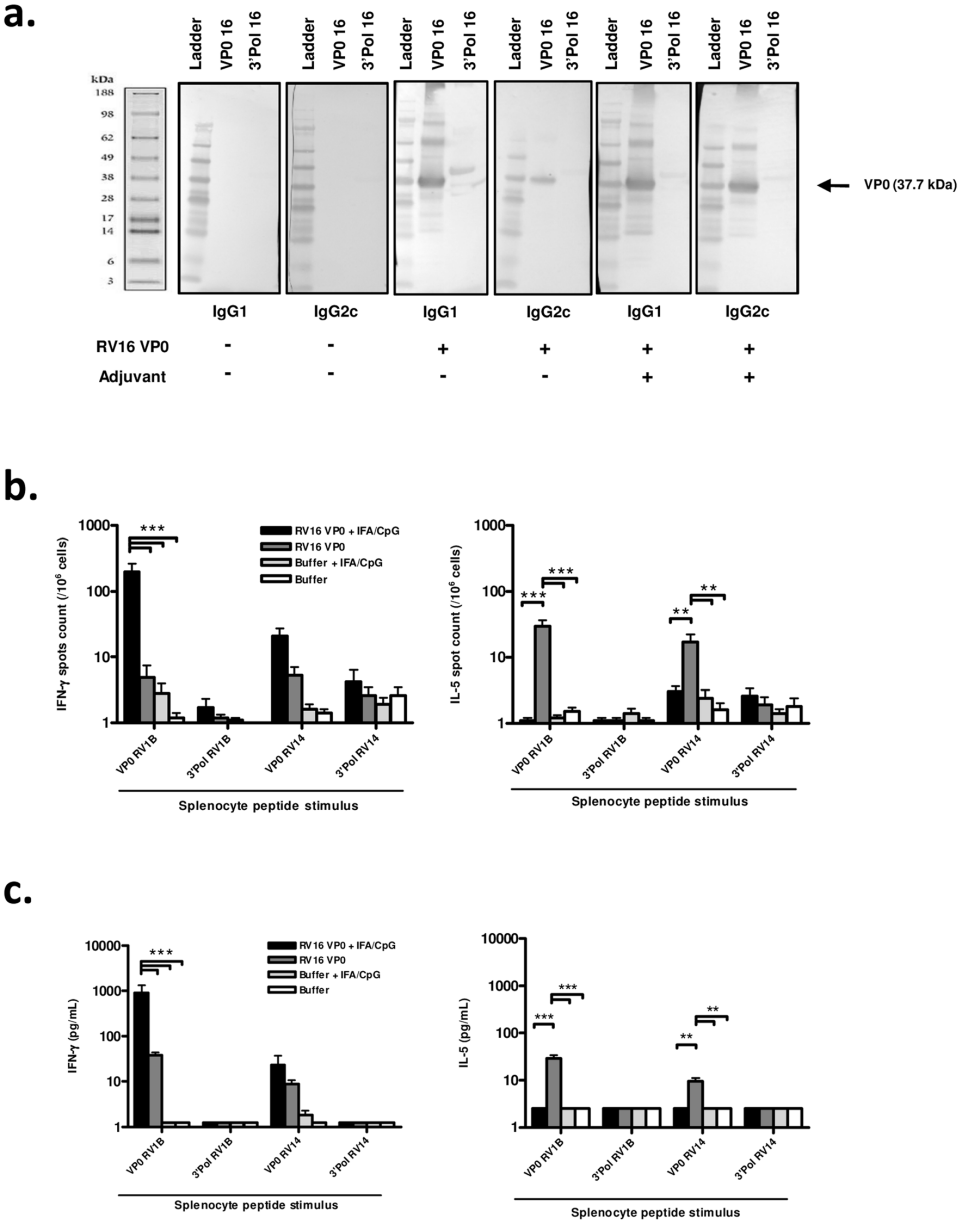
Immunization induces systemic, cross-serotype, type I immune responses. Mice were immunized subcutaneously with RV16 VP0 protein or buffer, with or without IFA/CpG adjuvant, as described. Spleens and serum were harvested 28 days post-immunization. (a) Serum IgG binding to (RV16 VP0 or control polymerase (3′ Pol)) viral proteins were assessed by western blot. (b & c) Splenocytes were stimulated with VP0 or Polymerase (3′ Pol) peptide pools as indicated and (b) IFN-γ and IL-5 producing cells were enumerated by ELISPOT assay and (c) supernatant FN-γ and IL-5 protein levels were measured by cytometric bead array. n = 10 mice/group ***P<0.001, **P<0.01.

We first assessed the immunogenicity of subcutaneously delivered RV16 VP0 protein. Analysis of antibody responses by western blot showed that RV16 VP0 - specific IgG was detectable in serum 28 days post-immunization (Fig. 1a). In mice immunized with VP0 protein alone, VP0-specific IgG1 and IgG2c, Th2 and Th1 associated IgG isotypes respectively, were detected.

To assess whether a Th1/Tc1 orientated response to infection is associated with improved disease outcome, we attempted to induce a Th1 skewed response to RV16 VP0 using a combination of incomplete freund's (IFA) and CpG adjuvants (IFA/CpG). The addition of IFA/CpG to the immunogen caused a more prominent IgG2c response (Fig. 1a).

Having established that RV16 VP0 is immunogenic, we next assessed the T cell response to immunization by measuring splenocyte cytokine production in response to stimulation with VP0, or control polymerase, peptides (described in Fig. S2). Stimulation with control polymerase peptides did not induce cytokine production (Fig. 1b,c). In both ELISPOT (Fig. 1b) and cytometric bead array (Fig. 1c) assays VP0 peptide pool stimulation induced IL-5, or both IL-5 and IFN-γ production by cells from mice immunized with VP0 protein alone, indicating a Th2 or mixed Th1/Th2 orientated response. As expected, the addition of IFA/CpG adjuvant to the immunogen caused a near complete suppression of IL-5 and substantial increase in IFN-γ responses (IL-5 p<0.01, IFN-γ p<0.001 RV16 VP0+IFA/CpG vs RV16 VP0 treatment for VP0 peptide pool stimulation) (Fig. 1b,c). Importantly, splenocytes from major group A species RV16 VP0 protein immunized mice produced cytokines when stimulated with VP0 peptides based on minor group A species RV1B and major group B species RV14 sequences, indicating cross-serotype reactivity.

### Immunization enhances airway T cell responses to infection with a heterologous RV strain

**Figure 2:**
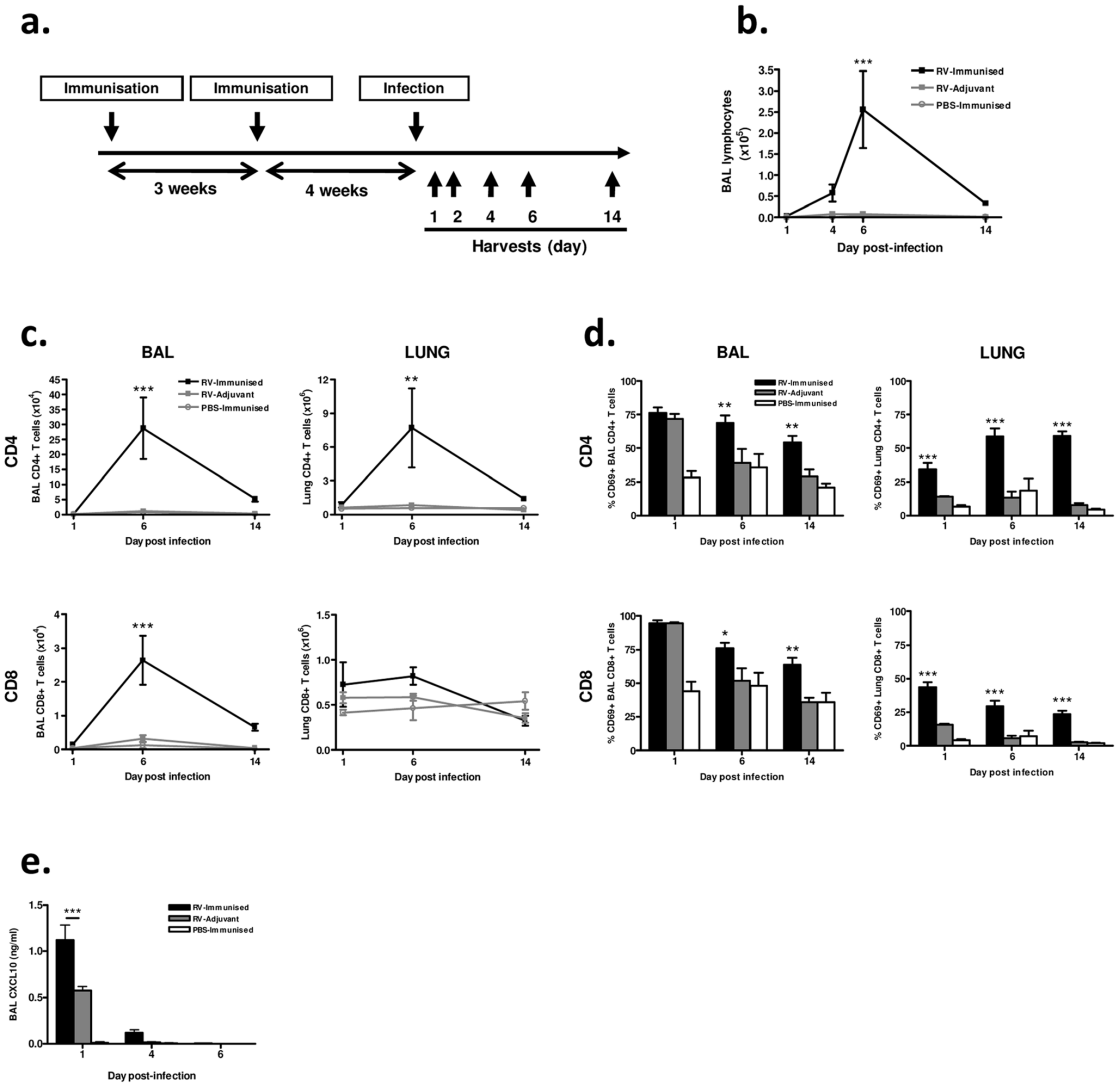
Immunization enhances airway lymphocyte responses to heterologous RV infection. (a) Mice were immunized subcutaneously with RV16 VP0 protein plus IFA/CpG adjuvant, or with IFA/CpG adjuvant only, and infected intranasally with RV1B (RV-Immunized, RV-Adjuvant) or sham PBS-challenged (PBS-Immunized). (b) Lymphocytes in BAL were counted by cytospin assay. (c) BAL and lung CD4+ and CD8+ T cells were enumerated and (d) their expression of the activation marker CD69 was assessed by flow cytometry. (e) CXCL10/IP-10 protein in BAL was measured by ELISA. n = 4 mice/group. Statistics indicated are for RV-immunized vs RV-adjuvant groups. ***P<0.001, **P<0.01, *P<0.05.

We next determined the effect of (major group, A) RV16 VP0 plus IFA/CpG immunization on responses to intranasal challenge with RV1B, a heterologous minor group A virus (Fig. 2a).

We observed no signs of clinical disease in animals which were immunized prior to infection consistent with our previous experience of mouse RV infections. Differential staining of bronchoalveolar lavage (BAL) leukocytes showed a significantly increased magnitude of lymphocyte response to infection in immunized and infected (RV-immunized) vs adjuvant treated and infected (RV-Adjuvant) mice (day 6 post-infection p<0.001) (Fig. 2b). To examine this enhanced lymphocyte response further, T cells in BAL and lung were analyzed by flow cytometry. CD4+ T cell numbers were substantially increased in both BAL and lung, and CD8+ T cell number was increased in BAL of RV-immunized vs RV-adjuvant treated mice on day 6 post-infection (p<0.01 BAL and lung CD4+ T cells, p<0.001 BAL CD8+ T cells) (Fig. 2c). The response in RV-immunized mice was dominated by CD4+ T cells whose number was ∼10-fold greater than CD8+ T cells by day 6 post-infection. In infected mice, the proportion of BAL and lung T cells expressing the activation marker CD69 was also significantly increased by immunization (RV-Immunised vs RV-adjuvant p<0.001 lung CD4+ and CD8+ T cells day 1–14, p<0.05 BAL CD4+ & CD8+ T cells d6 & d14)(Fig. 2d). Immunization-induced increases in T cell number were associated with enhanced levels of T cell chemokine CXCL10 (p<0.001 RV-Immunised vs RV-adjuvant at 24 hrs post infection)(Fig. 2e).

### Immunization induces antigen-specific lung Th1 responses to infection

**Figure 3:**
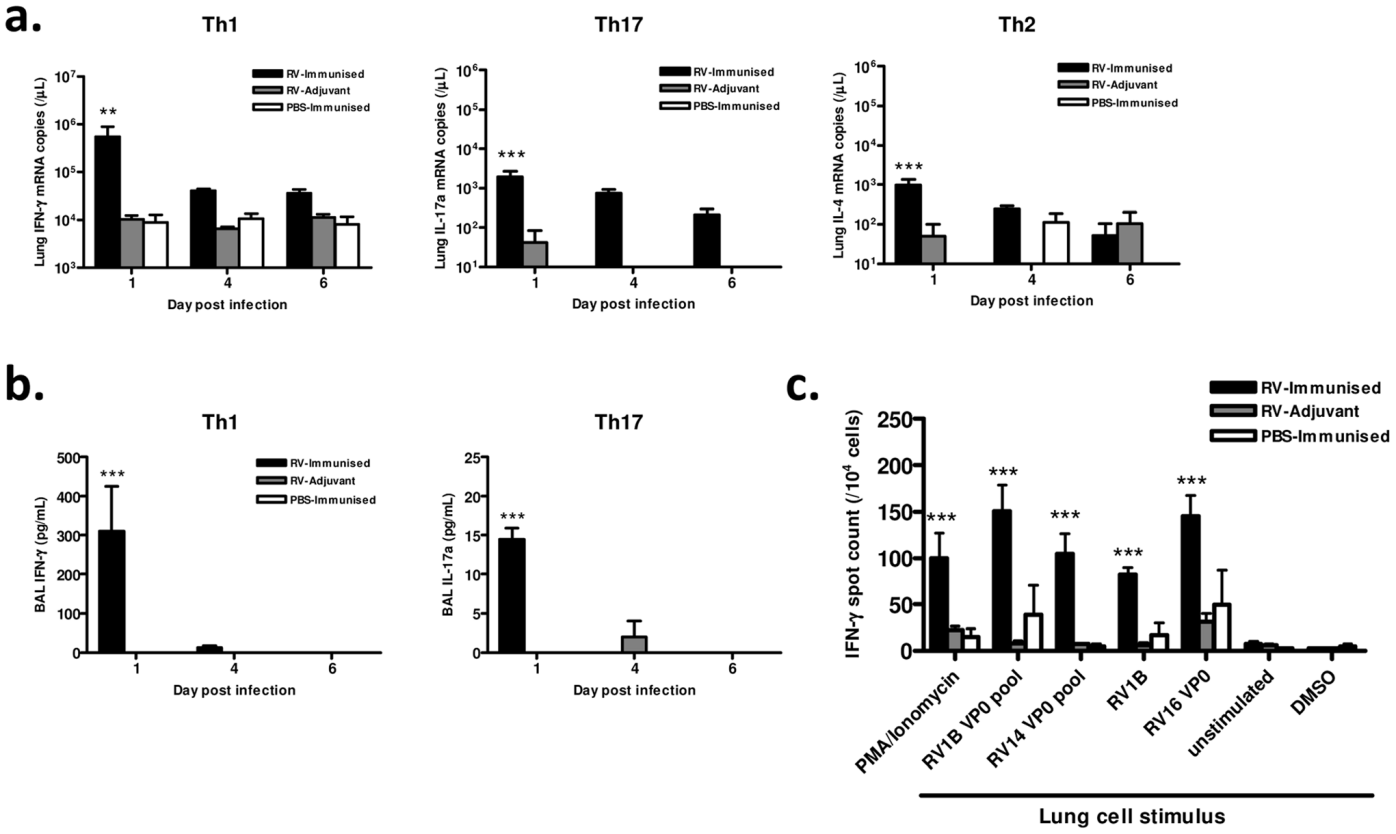
Immunization enhances lung Th1/Tc1 responses to heterologous RV infection. Mice were immunized subcutaneously with RV16 VP0 protein plus IFA/CpG, or with IFA/CpG adjuvant only and infected intranasally with RV1B or sham PBS-challenged, as described. (a) Lung tissue IFN-γ, IL-17a and IL-4 mRNA levels measured by Taqman qPCR. (b) T cell cytokine proteins in BAL measured by ELISA. (c) Lung cells harvested 6 days after intranasal challenge were incubated with the indicated stimuli and IFN-γ producing cells were enumerated by ELISPOT assay. n = 4 mice/group. Statistics indicated are for RV-immunized vs RV-adjuvant groups. ***P<0.001, **P<0.01.

We also examined the effect of immunization with RV16 VP0 on the polarity and antigen specificity of airway T cells after heterologous RV1B challenge. Immunization significantly increased the levels of signature Th1 (IFN-γ), Th17 (IL-17a) and Th2 (IL-4) cytokine mRNAs in lung tissue of RV1B challenged mice (p<0.01 RV-immunised vs RV-adjuvant at 24 hrs post-infection) (Fig. 3a). Consistent with the use of the Th1-promoting adjuvants, this response was dominated by IFN-γ in RV-immunized mice. IFN-γ and IL-17a protein were detected at 24 hrs post-infection only in immunized and challenged mice (p<0.001 vs RV-adjuvant treatment). IFN-γ again dominated with concentrations ∼20× higher than IL-17a (Fig. 3b). IL-4 protein was undetectable in BAL of all groups.

Since immunization generated cross-reactive, VP0-specific cells in the spleen (Fig. 1), we also determined if cross-reactive memory cells were recruited to the airways after infection by measuring IFN-γ production by antigen stimulated lung leukocytes using ELISPOT assays. The frequency of IFN-γ producing lung cells was greatest in mice both immunized and RV challenged (Fig. 3c). Stimulation with homosubtypic immunogen RV16 VP0, with heterotypic RV1B and RV14 VP0 peptide pools, and with live RV1B all induced similar IFN-γ responses (all viral stimuli p<0.001 RV-Immunised vs RV-adjuvant). RV16 VP0 immunization therefore induces cross-reactive Th1/Tc1 responses in the lung in response to RV1B challenge that are of significantly greater magnitude than with RV infection plus adjuvant treatment or immunization with sham infection (Fig. 3c).

### Immunization increases T cell responses to infection with a more distantly related RV serotype

RV16 and RV1B belong to different receptor binding groups (major and minor respectively), but are highly related at the nucleotide level [11] and the amino acid level (Fig. S1d) within VP0. To establish if immunization induces more broadly cross-reactive responses we therefore assessed responses to infection with the more distantly related [11] minor group A virus, RV29 (Fig. S1d).

**Figure 4:**
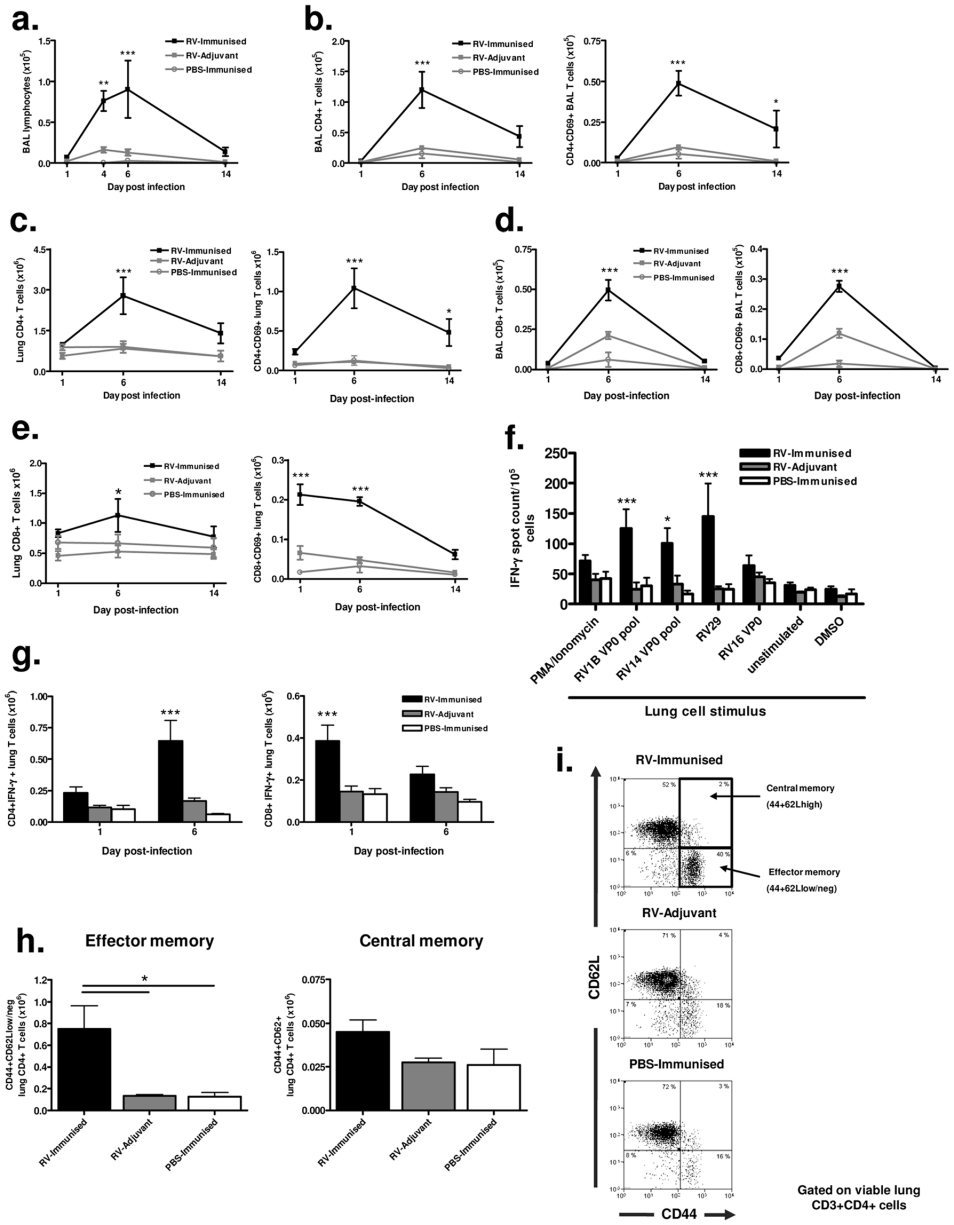
Immunization enhances effector and memory T cell responses to infection with a more distantly related RV. Mice were immunized subcutaneously with RV16 VP0 protein plus IFA/CpG or with IFA/CpG adjuvant only and infected intranasally with RV29 or sham PBS-challenged, as described. (a) Lymphocytes in BAL were counted by cytospin assay. (b & c) Total and CD69 expressing CD3+CD4+T cells in BAL (b) and lung (c) were enumerated by flow cytometry. (d & e) Total and CD69 expressing CD3+CD8+T cells in BAL (d) and lung (e) were enumerated by flow cytometry. (f) Lung cells harvested 6 days after intranasal challenge were incubated with the indicated virus, protein, peptide pool or control stimuli and IFN-γ producing cells were measured by ELISPOT assay. (g) Lung cells were stimulated with PMA and ionomycin and intracellular IFN-γ expression in CD4+ and CD8+ T cells was measured by flow cytometry. (h) Graphical data and (i) representative flow cytometry dot plots of CD62L and CD44 memory cell staining of lung CD4+ T cells on day 14 post-infection. n = 4 mice/group. Statistics indicated in a to g are for RV-immunized vs RV-adjuvant groups. ***P<0.001, **P<0.01, *P<0.05.

BAL cell staining revealed increased lymphocyte numbers in RV16 VP0 immunized and RV29 infected (RV-immunized) vs adjuvant treated and RV29 infected (RV-adjuvant) mice (p<0.01 day 4, p<0.001 day 7 post-infection)(Fig. 4a). Total and activated CD4+ (Fig. 4b & 4c) and CD8+ (Fig. 4d & 4e) T cell number in BAL and lung tissue were also significantly increased compared to infection or immunization treatments alone. Upon stimulation with RV antigens in ELISPOT assays, IFN-γ producing lung leukocyte frequency was greater in response to challenge serotype (RV29) stimulation in RV-immunized vs RV-adjuvant treated mice (p<0.001)(Fig. 4f). Similar increases were apparent after stimulation with RV1B (p<0.001) and RV14 (p<0.05) derived VP0 peptide pools, again indicating cross-serotype reactivity. We also determined lung T cell-specific IFN-γ production by intracellular flow cytometry staining and observed early (day 1) increases in CD8+ and later (day 6) increases in CD4+ T cells expressing IFN-γ in RV-immunized vs RV-adjuvant, or PBS-immunized treatment groups (RV-immunised vs RV-adjuvant p<0.001) (Fig. 4g).

### Immunization enhances lung memory T cell responses to heterologous virus infection

Significantly increased numbers of activated CD4+ T cells persisted in the lungs of immunized and RV infected mice on day 14 post-infection (Fig. 4c). To determine if this represented enhanced generation of local T cell memory we performed flow cytometric staining for memory markers on lung CD4+ T cells. The proportion and absolute number of CD4+ T cells with a CD44+CD62L^low^, effector memory, phenotype was significantly higher in RV29 infected and RV16 VP0 immunized mice compared to either treatment alone (p<0.05). However, no differences were observed between groups in CD44+CD62L^high^ central memory cells (Fig. 4h & 4i).

### Immunization enhances neutralizing antibody responses to heterologous virus infection

As neutralizing antibodies are believed important in protection against RV infection, we next investigated the effect of immunization on generation of humoral immune responses by measuring serum and BAL immunoglobulin binding to RVs, and the ability of sera to neutralize RV infection *in vitro*.

ELISA binding assays showed that immunization with RV16 VP0 in the absence of RV infection weakly induced RV29 and RV1B binding antibodies (Fig. S3a–d). The cross-reactivity of antibodies induced by RV16 VP0 immunization against multiple virus serotypes was also shown by Western Blot (Fig. S3e). When combined with RV1B or RV29 infection *in vivo*, immunization generated more rapid and greater magnitude of RV-specific serum and BAL IgG responses, and BAL IgA responses, than RV-adjuvant treatment (Fig. S3a–d), indicating that immunization also boosts antibody responses upon subsequent heterotypic RV infection.

**Figure 5:**
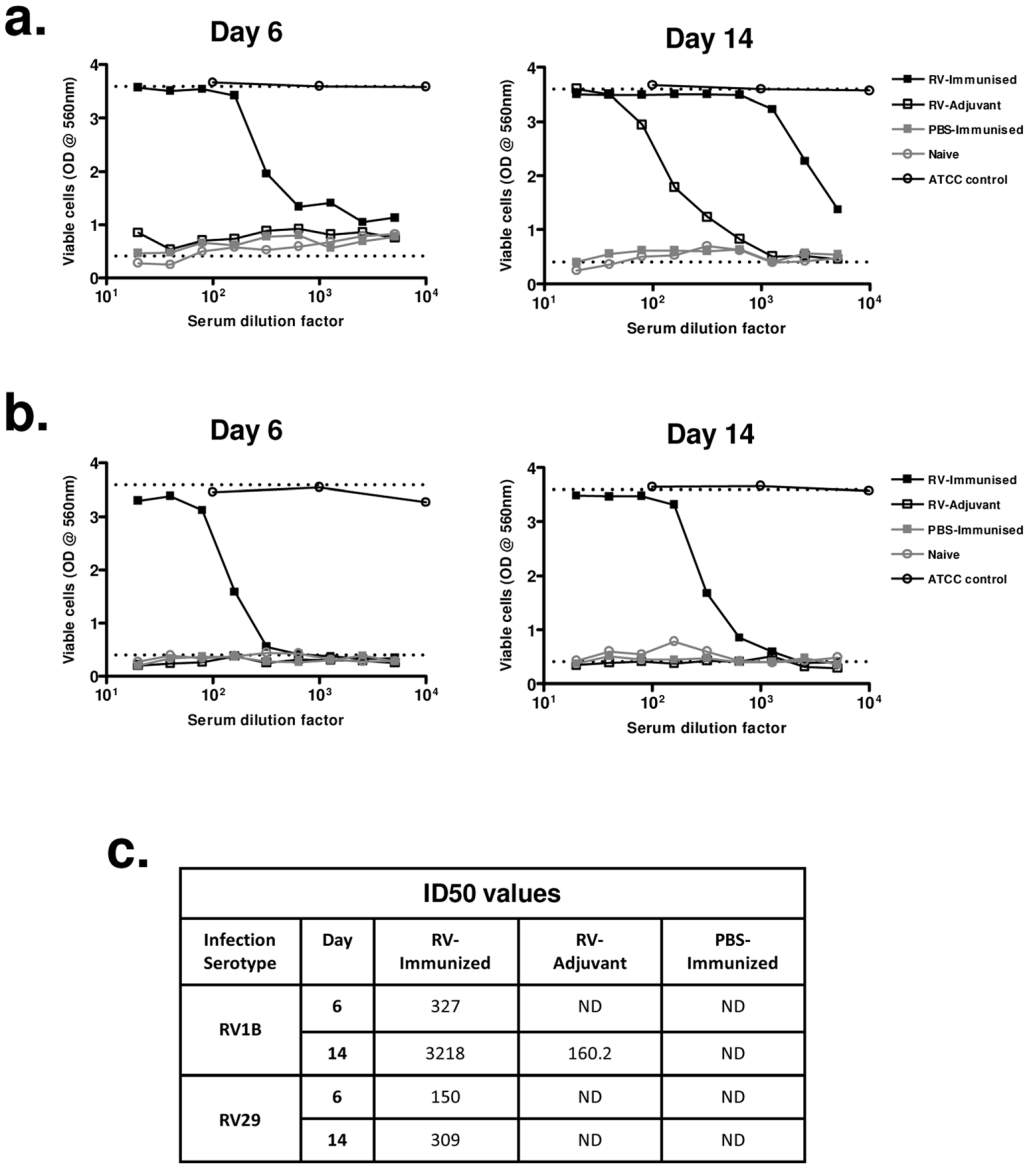
Immunization enhances and accelerates the generation of neutralizing antibodies to a heterologous infecting virus. Mice were immunized subcutaneously with RV16 VP0 protein plus IFA/CpG or with IFA/CpG adjuvant only and infected intranasally with RV1B, RV29 or sham PBS-challenged as described. Sera were assayed for their ability to prevent cytopathic effect caused by the same RV serotype administered for *in vivo* infection, using a crystal violet HeLa cell neutalization assay. (a) Neutralization of RV1B cytopathic effect by sera from RV1B-infected or PBS-challenged mice. (b) Neutralization of RV29 cytopathic effect by sera from RV29 infected or PBS challenged mice. Top dotted lines; serum only (uninfected) controls. Bottom dotted lines; virus infected (no serum) control. Open circles are ATCC reference guinea pig anti-sera. Data points represent sera pooled from 4 mice/treatment group. (C) Serum 50% inhibition dilution (ID_50_) values for RV1B and RV29 neutralization. ND; not detected.

We next investigated if enhanced heterotypic antibody responses included boosting of neutralizing activity. Immunization with RV16 VP0 alone did not induce neutralizing antibodies in uninfected mice (Fig. 5a,b). Neutralization of the infecting serotype virus was observed with day 14 post-infection sera of mice treated with adjuvant and infected with RV1B (Fig. 5a), but this was not observed for RV29 (Fig. 5b), suggesting that the neutralizing antibody response to RV in the mouse is either weak or absent. Prior immunization of RV challenged mice however induced both a more rapid induction (day 6) and greater peak titer of neutralizing antibodies (RV1B infection: 50% inhibition dilution [ID50] day 14 RV-immunized 1∶3218 vs RV-adjuvant 1∶160) (Fig. 5a–c). Antibodies induced by RV16 VP0 immunization only neutralized the *in vivo* infecting RV serotype (data not shown).

These data indicate that immunization with RV16 VP0 is capable of substantially enhancing neutralizing antibody responses to *in vivo* infection with heterologous RVs.

### Immunization accelerates virus clearance

**Figure 6:**
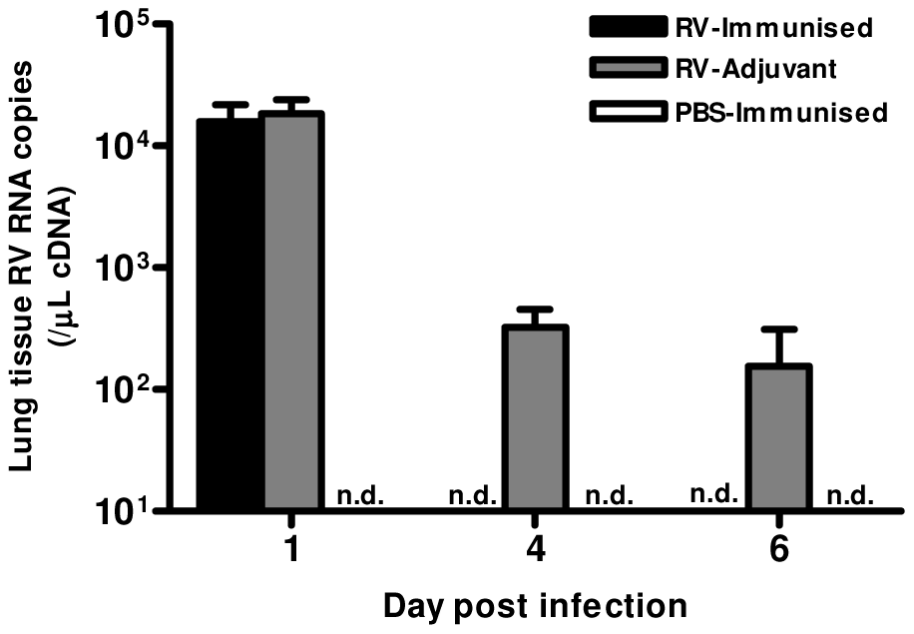
Immunization accelerates virus clearance. Mice were immunized subcutaneously with RV16 VP0 protein plus IFA/CpG or with IFA/CpG adjuvant only and infected intranasally with RV1B or sham PBS-challenged. RV RNA in lung tissue was measured by Taqman qPCR. n = 4 mice/group. n.d., not detected.

Finally, we determined whether the Th1 and neutralizing antibody responses induced by immunization conferred any benefit on virus control. Immunization resulted in more rapid virus clearance, as RV1B RNA was undetectable on days 4 & 6 in RV-immunized but not in adjuvant treated mice (Fig. 6).

## Discussion

The unmet medical need attributable to RV infections is enormous but serotypic heterogeneity represents a major barrier to the development of an RV vaccine. We therefore identified regions of the RV polyprotein which are highly conserved amongst RVs to select potential constituents of a broadly cross-reactive subunit vaccine and tested their efficacy in a mouse model. We found that domains of the VP4 and VP2 (VP0) capsid proteins were highly conserved across A and B species RVs.

Immunization with recombinant RV16 VP0 protein increased the magnitude of airway T cell, especially CD4+ T cell, responses to infection consistent with the recruitment to and expansion of immunization-induced memory T cells in the airways. Although the CD4+ T cell dominance of this response contrasts with the prominent CD8+ CTL responses characteristic of other respiratory virus infections [24], [25], [26], there is evidence to suggest this is representative of naturally occurring RV infection [20], [27].

CD4+ T cells provide B cell help and can also possess direct cytotoxic effector function similar to CD8+ CTL and could therefore have both direct and indirect roles in RV control [28], [29], [30], [31].

The observed increases in airway T cell number in immunized and infected mice might in part be explained by the enhanced levels of the T cell recruiting chemokine CXCL10 measured in BAL 24 hrs after infection. Locally induced or systemically transferred memory T cells have previously been shown to increase airway innate immune mediators after influenza challenge via both IFN-γ dependent and independent mechanisms [32]. CXCL10 is an interferon inducible gene and in our studies the increase in CXCL10 might be explained by the enhanced levels of IFN-γ in the lungs of immunized and infected mice at the same timepoint after infection.

There is limited data available regarding T cell polarization during RV infections in humans. In the mouse model little T cell cytokine response was measurable in the airways of infected and adjuvant treated mice, but by combining Th1 promoting adjuvants with the VP0 immunogen we observed a strong type I response to RV challenge and an acceleration of rhinovirus clearance. This is the first clear evidence that such enhancement of type I polarized T cell responses to RV provides benefit in terms of virus control [19], [22], [33]. In addition, asthmatics are a major target group for RV vaccination and Th1/Tc1 responses may also suppress type 2 responses which are associated with increased disease severity during experimental RV-induced disease exacerbations in atopic asthmatics [33], a hypothesis which can now be tested by utilising the mouse RV-induced asthma exacerbation model we have described previously [23]. A key requirement for an RV vaccine is broad cross-reactivity against the ∼150 strains. Human memory CD4+ T cells specific for conserved influenza proteins have been demonstrated to be cross-subtype responsive [17], [18] and we hypothesized that immunization with conserved RV proteins might induce similarly cross-reactive cells. We found that RV16 VP0 immunization induced systemic T cells that were responsive to VP0 peptides from heterologous group A and group B RV serotypes. Following subsequent challenge, cells recovered from the lungs were reactive to the RV16 derived immunogen, to heterologous group A live viruses with which mice were infected and to group B RV VP0 peptides. This cross-reactivity likely represents the recognition of conserved epitopes within VP0, primarily by CD4+ T cells given their greater expansion. Whether this cross-reactivity will be similarly evident in human populations with diverse MHC is not known but a previous study encouragingly showed that VP2 peptides can induce cross-haplotype responses in mice [21]. Further, whilst these studies provide proof of concept for the generation of cross-reactive T cells to RVs, further studies should determine if similar cross-reactivity is seen for the ∼100 other known RV serotypes. Likewise the large number of genetically defined C species RVs which are to date not well characterized [13]. Whether vaccine induced enhancement of Th1 cell responses to RV will prove a safe strategy for preventing RV induced disease awaits confirmation in a clinical trial. However, influenza vaccines are already licenced which use adjuvants which promote strong CD4+ T cell responses and have been shown to be safe [34], [35].

Immunization also induced IgG antibodies which bound multiple RV serotypes and following subsequent infection, enhanced heterologous infection serotype specific antibody levels in serum and BAL. Notably, this included a BAL IgA response which as we have shown previously [36] is otherwise weak or absent after a single infection in this model. Importantly, immunization with RV16 VP0 also enhanced neutralizing antibody responses to infection with the heterosubtypic viruses RV1B and RV29. The fact that generation of neutralizing antibody was dependent upon infection suggests that the effect of immunization on production of serotype-specific neutralizing antibodies following subsequent infection results from B cell help provided by broadly responsive immunization-induced T cells. Enhancement of both the speed and magnitude of antibody responses may provide benefit in terms of accelerating virus clearance and reducing duration of disease caused by naturally occurring infections with virus strains heterologous to that upon which the sequence of the immunogen is based.

Consistent with a role for immunization-induced responses in enhancing virus control, we found that viral RNA was cleared more rapidly from the lungs of immunized mice after subsequent virus infection. This effect was more evident at later stages of infection, which is likely attributable to the fact that virus replication in this mouse model is short-lived compared to human infection, lasting only around 24 hrs [23], [33], [37] and therefore before enhanced T cell responses are apparent. The fact that T cell and antibody responses were able to speed virus clearance in a mouse model where replication is short lived suggests however that in man, where replication is much more robust and of longer duration [33], [37], the magnitude of benefit might be substantially greater.

Pre-existing neutralizing antibodies to RVs provide protection against infection and symptoms in humans [6], [7] and in addition to accelerating virus clearance during the first naturally acquired infection with a given serotype, enhanced neutralizing antibody responses may provide better and more durable protection against future RV infections. Likewise the enlarged effector memory T cell pool in immunized persons, because local memory T cells are likely to respond rapidly to secondary challenge and are proposed to possess more potent anti-viral function than systemic memory cells [38], [39]. Immunization with VP0 may therefore generate serotype specific protective humoral and cross-reactive lung T cell memory responses to natural infection. Because RV infections are frequent throughout life, typically comprising 8–10 per year in young children and 2–5 per year in adults [40], natural infection following immunization could result in protection against a broad range of previously unseen RVs.

In summary, immunization with a recombinant RV capsid protein enhanced airways Th1 cell and airways and systemic antibody responses to infection with heterologous virus serotypes. Immunization also accelerated virus clearance. This study therefore provides proof of principle for a broadly cross-reactive subunit vaccine for RV infections.

## Materials and Methods

### Ethics statement

All animal studies were conducted according to UK home office legislation (Animals (Scientific Procedures) Act 1986), project licence number PPL 70/7234, or under approval of the Sanofi Pasteur Animal Care Committee protocol numbers F.DI.RVI005.Ms, F.DI.RVI006.Ms and F.DI.RVI007.Ms.

### Identification of conserved sequences

The design of the VP0 immunogen was based on linear sequence conservation amongst RVs. All RV sequences were retrieved from the National Center for Biotechnology Information (NCBI) Genbank database on August 23, 2007 and sequence alignments were generated for all available complete polyproteins from HRV-A and HRV-B using the MUSCLE algorithm [41]. This included 136 polyprotein sequences across 74 A species serotypes and 51 sequences across 25 B species serotypes to take account of variability both between serotypes and between different field strains within serotypes. A phylogenetic tree was elaborated using the maximum likelihood method from the Seaview application [42] and bootstrap values were calculated to assess the robustness of the nodes. A global consensus sequence was generated from the alignments using the Jalview application [43]. Global consensus sequences were extracted from each alignment and frequency of occurrence for each major amino acid was calculated (Fig. S1 a,b).

### Expression and purification of antigens

The VP0 nucleotide sequence was optimized for *E. coli* expression and synthesized (Life Technologies, Saint Aubin, France). Antigen was expressed as a recombinant protein fused to a SUMO tag using the pET-SUMO vector (Invitrogen, Saint Aubin, France). The Overnight Express Autoinduction System 1 (EMD Millipore, France) was used with BL21λDE3 *E. coli* transfected with the pET-SUMO plasmid encoding RV16 VP0. As it was expressed into the insoluble fractions as inclusion bodies, purification was then performed according the manufacturer recommendations (Invitrogen) adapted for insoluble proteins.

Briefly, SUMO-fused proteins extracted with Tris/NaCl buffer containing 8M Urea were loaded onto Nickel sepharose columns (Pharmacia) for Immobilized Metal Affinity chromatography (IMAC). Purification was performed by applying an imidazole gradient to the column. Recombinant proteins eluted into the 250 mM imidazole fractions were further dialysed against a digestion buffer (Tris 20 mM, NaCl 150 mM pH 8.0 containing 2M Urea) to cleave the SUMO moiety by the SUMO ULP-1 protease. The RV16 VP0 obtained after digestion was applied onto a second Nickel sepharose column to remove the SUMO moiety, the non-cleaved protein and the protease-containing His tag (Fig. S1e). The cleaved RV16 VP0 obtained after the second purification step was further dialysed against Tris/NaCl buffer (Tris 20 mM, NaCl 150 mM, Arginine 0.5 M, pH 8.0).

Peptide pools for RV1B and RV14 were generated for the VP0 and 3′ polymerase regions. Peptides were synthesized and purified commercially (JPT, Germany). Peptides were 15mers overlapping by 11 amino acids, with each pool comprising approximately 40 peptides. The sequences upon which the respective peptide pools are based are presented in Figure S2.

### RV propagation

RV serotype 1B and 29 for *in vivo* studies were propagated in H1 HeLa cells (American Type Tissue Culture Collection (ATCC) ref CRL-1958) and purified and titrated as described previously [23]. RV stocks were originally obtained from the ATCC. A purified, uninfected HeLa cell lysate preparation was generated as a control for virus-specific immunoglobulin assays.

### Mice

6–8 week old, wild type, female C57BL/6 mice were purchased from Charles River Laboratories (UK, or Saint Germain sur l'Arbresle, France) and housed in individually ventilated cages.

### *In vivo* protocols

For immunogenicity experiments (Fig. 1), mice were immunised subcutaneously (s.c.) on days 0 and 21 with 10 µg RV16 VP0 protein, Incomplete Freund's and CpG (IFA/CpG) adjuvant (10 µg CpG 1826 (MWG Eurofins, Germany) and 100 µL IFA), or with adjuvant alone. Further controls received protein buffer (Tris 20 mM, NaCl 150 mM, Arginine 0,5 M pH 8,0) with or without IFA/CpG adjuvant. Mice were culled on day 49.

For RV challenge studies mice were immunised s.c. on days 0 and 21 with a solution containing; 10 µg RV16 VP0 protein, 10 µL CpG oligonucleotide (100 µM ODN 1826) and 40 µL IFA (Sigma-Aldrich, UK) in sterile PBS, or adjuvant alone. On day 51, mice were challenged intranasally with 5×10^6^ TCID_50_ RV serotype 1B or 29, or mock challenged with PBS, and were culled at the indicated timepoints.

### Tissue harvesting and processing

Bronchoalveolar lavage (BAL) was performed and processed as previously described [23]. For lung leukocyte analyses, tissue was homogenized using the GentleMACS tissue dissociator (Miltenyi Biotech, UK) and homogenized tissue was digested in RPMI medium containing 1 mg/ml collagenase type XI and 80units/mL bovine pancreatic Dnase type IV (both Sigma-Aldrich). Red cells were lysed with ACK buffer. For RNA extraction, an apical lobe of the right lung was excised and stored in RNAlater stabilization buffer (Qiagen, UK). Splenocytes were isolated by manually homogenizing spleens through a cell strainer and treating with Hybri Max Red Blood Cell Lysing Buffer (Sigma- Aldrich). Blood was collected from the carotid arteries into ‘microtainer’ serum separation tubes or Vacutainer Vials (both BD Biosciences) and serum was separated by centrifugation.

### Cytospin assay

BAL cells were spun onto slides, stained and lymphocytes were counted as previously described [23]. Counts were performed blind to experimental conditions.

### Flow cytometry

1–10×10^5^ lung or BAL cells were stained with ‘live/dead fixable dead cell stain’ (Invitrogen) and incubated with anti-mouse CD16/CD32 (FC block; BD biosciences). Directly fluorochrome-conjugated antibodies specific for CD3-Pacific Blue (clone 500A2), CD4-APC (clone RM4-5), CD8-PE (clone 53-6.7), CD69-FITC (clone H1.2F3), CD62L-PE (clone MEL-14) and CD44-FITC (clone IM7) (all BD biosciences) were added directly. Cells were fixed with 2% formaldehyde. For intracellular staining, lung cells were stimulated for 4 hrs in media containing 50 ng/mL Ionomycin, 500 ng/mL PMA (Both Sigma Aldrich) and golgi transport inhibitor (Golgi Stop, BD Biosciences). Cells were then surface stained as described, permeablised with 0.5% (w/v) saponin (Sigma-Aldrich) and stained with fluorochrome conjugated anti-IFN-γ-FITC (clone XMG1.2, BD biosciences).

Flow cytometry data was acquired using CyanADP (Dako, USA) and FACSCanto (BD biosciences) cytometers and analysed using Summit software (Dako, USA).

### ELISA

Cytokine and chemokine proteins in BAL were assayed using protocols and reagents from *Duoset* ELISA kits (R&D systems).

RV-specific IgG and IgA were measured using in-house assays as described previously [36]. 96 well plates were coated overnight with purified RV1B or RV29, as used for in vivo infections, and blocked with 5% milk in PBS-0.05% tween 20. Samples were pooled for each treatment group/timepoint, diluted as indicated in 5% milk blocking solution and plates were incubated for a further 2 hrs at room temperature. Detection antibodies were biotinylated rat anti-mouse IgG1 (clone A85-1), IgG2a/c (clone R19-15) and IgA (clone C10-1) (all BD biosciences) diluted in PBS 1% BSA. Plates were then incubated with spreptavidin-HRP followed by TMB substrate (both Invitrogen) and reactions were stopped by addition of 1M H_2_SO_4_ For analysis of IgA in BAL, IgG was first depleted by incubation with protein G sepharose beads (Sigma-Aldrich). Antibody binding to HeLa cell lysate control coated wells was measured in parallel in all assays and values were subtracted from those of virus coated wells during analysis.

### Cytometric Bead Array (CBA)

4×10^5^ splenocytes per well were distributed in 96 well plates and stimulated with 1 µg/mL of RV peptide pools. Supernatants were harvested after 3 days at 37°C. IL-5 and IFN-γ concentrations were measured using the mouse Th1/Th2 cytokine kit (BD Biosciences) and a Facscalibur cytometer (Becton Dickinson). Data was analyzed on FCAP Array software (Becton Dickinson).

### ELISPOT

Assays were performed in 96 well multiscreen HA plates (Millipore) coated with purified anti-mouse IFN-γ or IL-5 (BD biosciences). After blocking, 1 or 2×10^5^ lung cells were added, followed by medium containing RV or control stimuli (RV16 VP0 protein (25 µg/mL), live RV1B (2×10^6^ TCID50/mL), RV peptide pools (1 or 4 µg/mL), DMSO peptide pool control, PMA/Ionomycin (50/500 ng/mL)). Plates were incubated for 18 hrs or 3 days at 37°C. Detection antibodies were biotinylated rat anti-mouse IFN-γ or IL-5 (BD biosciences). Plates were subsequently incubated with streptavidin-horseradish peroxidase (Southern Biotech) or extravidin alkaline phosphatase (Sigma-Aldrich) followed by AEC or NBT/BCIP substrate (both Sigma-Aldrich), respectively. Reactions were stopped with water.

### Western blots

In immunogenicity experiments (Fig. 1), IgG responses were analyzed by Western blot of pooled sera. 2 µg of recombinant viral protein and molecular weight standard (SeeBluePlus2, Invitrogen) were run on a 4–12% polyacrylamide SDS gel (Invitrogen). Protein was transferred onto a nitrocellulose membrane (Bio-Rad, USA) and blocked with 5% milk in PBS 0.05% Tween 20. Membranes were probed with (1 in 200) diluted pooled mouse sera followed by HRP-conjugated goat anti-mouse IgG (Jackson ImmunoResearch, UK). Blots were developed colorimetrically using 4-chloro-1-naphthol Opti-4CN substrate (Bio-Rad).

For the study of antibody cross-reactivity (Fig. S3) blots were performed as described but with 1.25 µg virus protein (*in vivo* inoculum) or 12.5 ng recombinant RV16 VP0. Detection antibody was goat anti-mouse IgG (Santa Cruz biotechnology, USA) and blots were developed using ECL (GE Healthcare, UK).

### Neutralisation assays

Neutralisation of RV was measured in Ohio HeLa cells (UK Health Protection Agency General Cell Collection catalogue number 84121901). Sera for given treatment groups/timepoints were pooled and incubated with purified RV at room temperature with shaking for 1 hr, before addition of HeLa cells and further incubation at 37°C for 48–96 hrs. Protection from CPE was measured by crystal violet cell viability assay whereby cells were stained with 0.1% (w/v) crystal violet, washed with water, air dried and crystal violet was solubilised with 1% SDS. Absorbance was measured at 560 nm.

### Statistical analysis

Graphical data is expressed as mean +/− SEM, representative of at least 2 independent experiments. For all data differences between treatment groups were assessed by one or two way ANOVA and if significant (*P*<0.05) individual differences were identified using bonferroni post-tests.

## Supporting Information

Figure S1 **Immunogen design.** (a) Mean linear amino acid sequence conservation amongst A (black line) and B (grey line) group RVs. The mean conservation level was calculated at each position as a sliding window of 30 amino acids in length. (b) Consensus amino acid sequence for the VP0 protein of all available RVs showing percentage conservation at each amino acid position. (c) Sequence of the RV16 VP0 immunogen. (d) Amino acid alignment for the VP0 protein of RV strains used in peptide generation and *in vivo* infections, with consensus sequence. (e) SDS-PAGE gel showing final step purification of the RV16 VP0 immunogen. MW, molecular weight marker. Lane a, SUMO VP0 protein after SUMO ULP-1 protease digestion. Lane b, cleaved HRV 16 VP0 protein after IMAC purification. Lane c, eluted SUMO moiety, non-cleaved protein and protease containing His tag.
(TIF)

Figure S2 **Peptide pools Amino acid sequences of VP0 and 3′ polymerase regions of RV1B and RV14 polyproteins.** Those sequences used for generation of VP0 and polymerase (3′Pol) peptide pools, as described in methods, are underlined.
(TIF)

Figure S3 **Serum and BAL antibody responses.** (a–d) Mice were immunized subcutaneously with RV16 VP0 protein plus IFA/CpG, or with IFA/CpG adjuvant alone and infected intranasally with RV1B, RV29 or sham infected with PBS, as described. Sera and BAL were harvested at 6 and 14 days post-infection, pooled and assayed for IgG and IgA binding to virus inoculum preparations. (a) Serum and (b) BAL RV1B binding in RV1B-infected or PBS-challenged mouse sera. (c) Serum and (d) BAL RV29 binding in RV29-infected or PBS-challenged mouse sera. (e) Mice were immunized twice with RV16 VP0 protein plus IFA/CpG adjuvant subcutaneously and serum was harvested 6 weeks after immunization. Serum IgG binding to RV16 VP0 immunogen or to RV1B, RV29 and RV16 was assessed by Western blot. HeLa; virus culture cell lysate control. VP0; viral VP0 protein band estimated by molecular weight.
(TIF)
