# Supplementary figures and images for: Cross-Serotype Immunity Induced by Immunization with a Conserved Rhinovirus Capsid Protein
Source: PLoS Pathog. 2013 Sep 26;9(9):e1003669. doi: 10.1371/journal.ppat.1003669 (PMC3784482; doi:10.1371/journal.ppat.1003669)

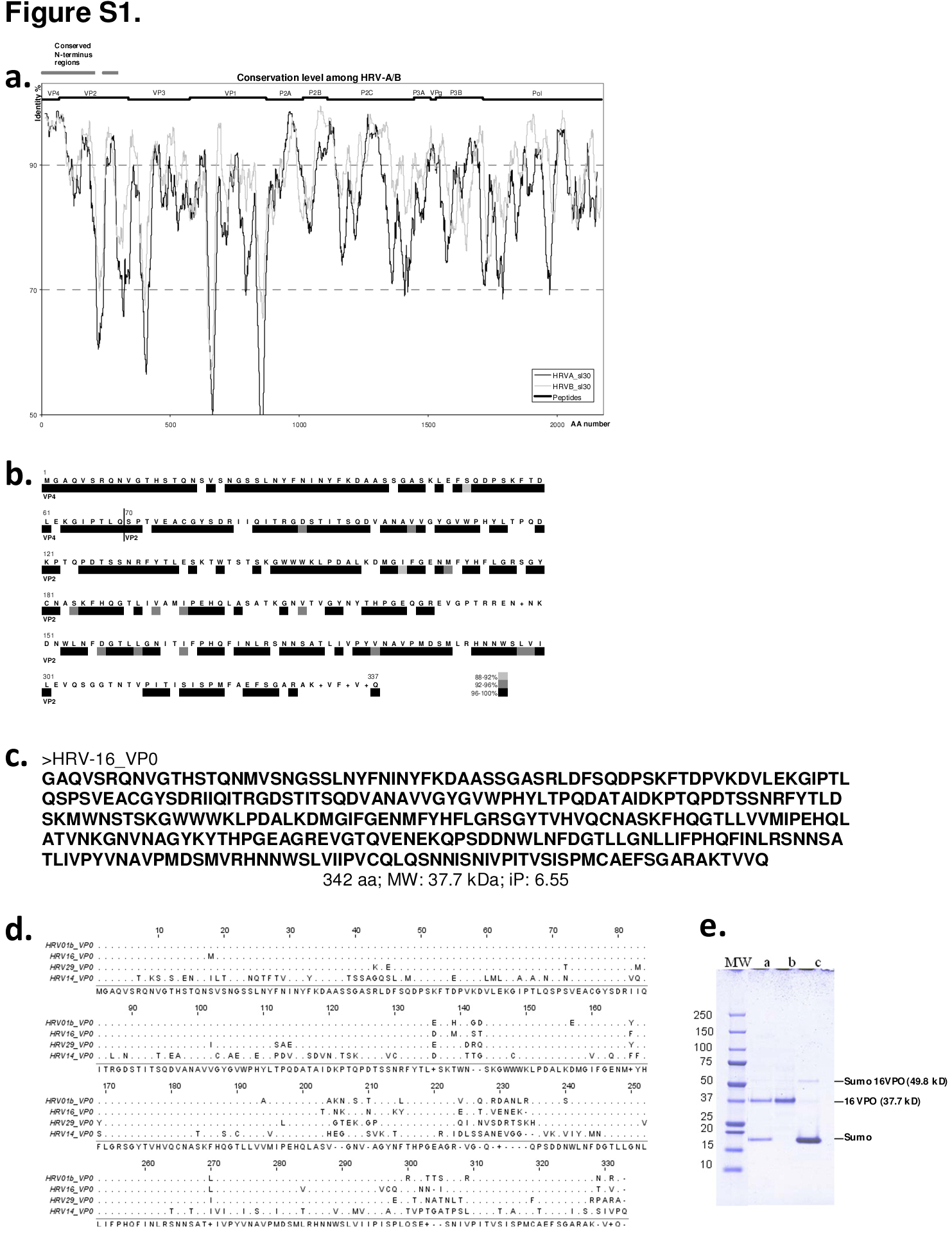

Supplement: Figure S1 — Immunogen design. (a) Mean linear amino acid sequence conservation amongst A (black line) and B (grey line) group RVs. The mean conservation level was calculated at each position as a sliding window of 30 amino acids in length. (b) Consensus amino acid sequence for the VP0 protein of all available RVs showing percentage conservation at each amino acid position. (c) Sequence of the RV16 VP0 immunogen. (d) Amino acid alignment for the VP0 protein of RV strains used in peptide generation and in vivo infections, with consensus sequence. (e) SDS-PAGE gel showing final step purification of the RV16 VP0 immunogen. MW, molecular weight marker. Lane a, SUMO VP0 protein after SUMO ULP-1 protease digestion. Lane b, cleaved HRV 16 VP0 protein after IMAC purification. Lane c, eluted SUMO moiety, non-cleaved protein and protease containing His tag. (TIF) [file ppat.1003669.s001.tif]

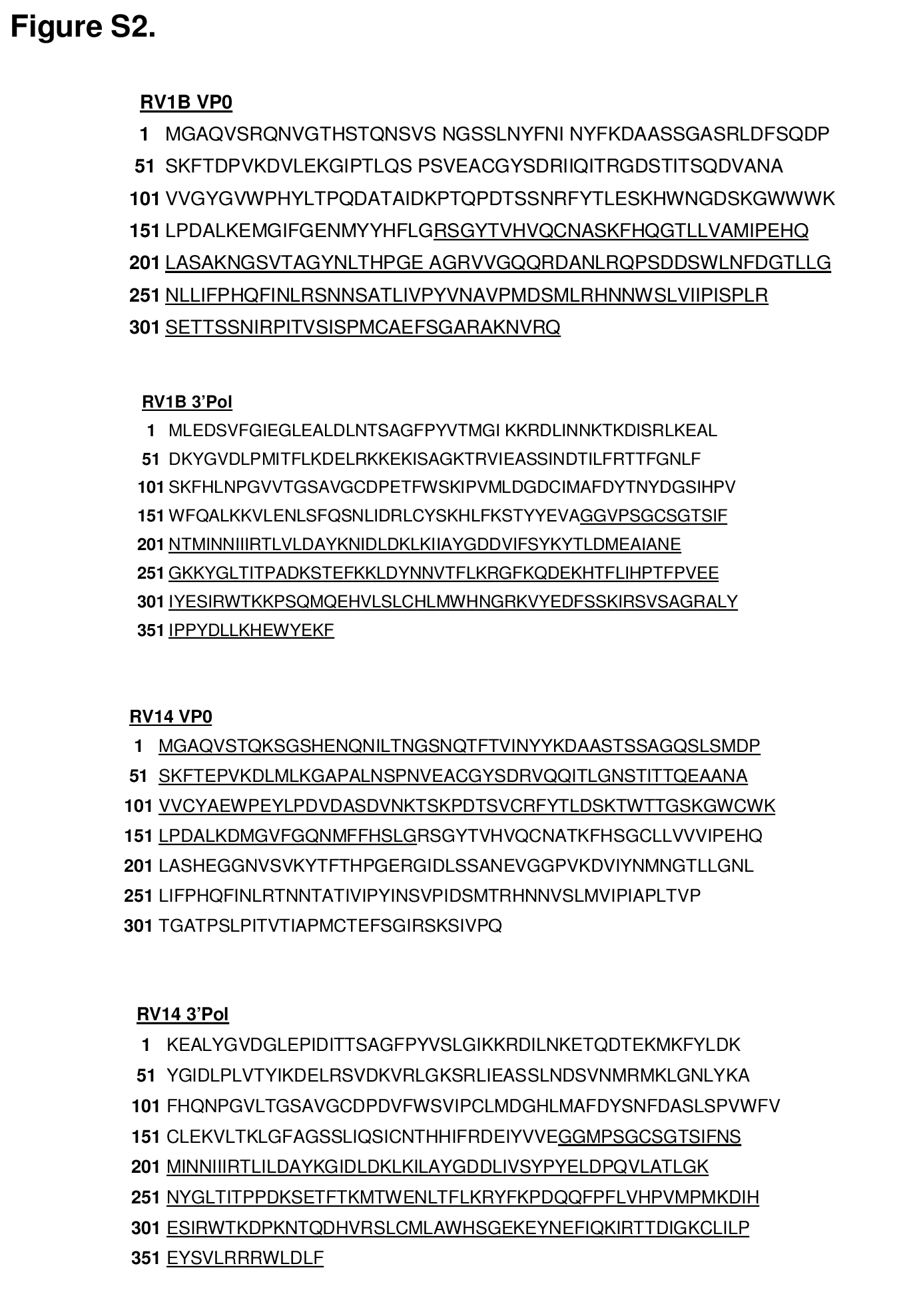

Supplement: Figure S2 — Peptide pools Amino acid sequences of VP0 and 3′ polymerase regions of RV1B and RV14 polyproteins. Those sequences used for generation of VP0 and polymerase (3′Pol) peptide pools, as described in methods, are underlined. (TIF) [file ppat.1003669.s002.tif]

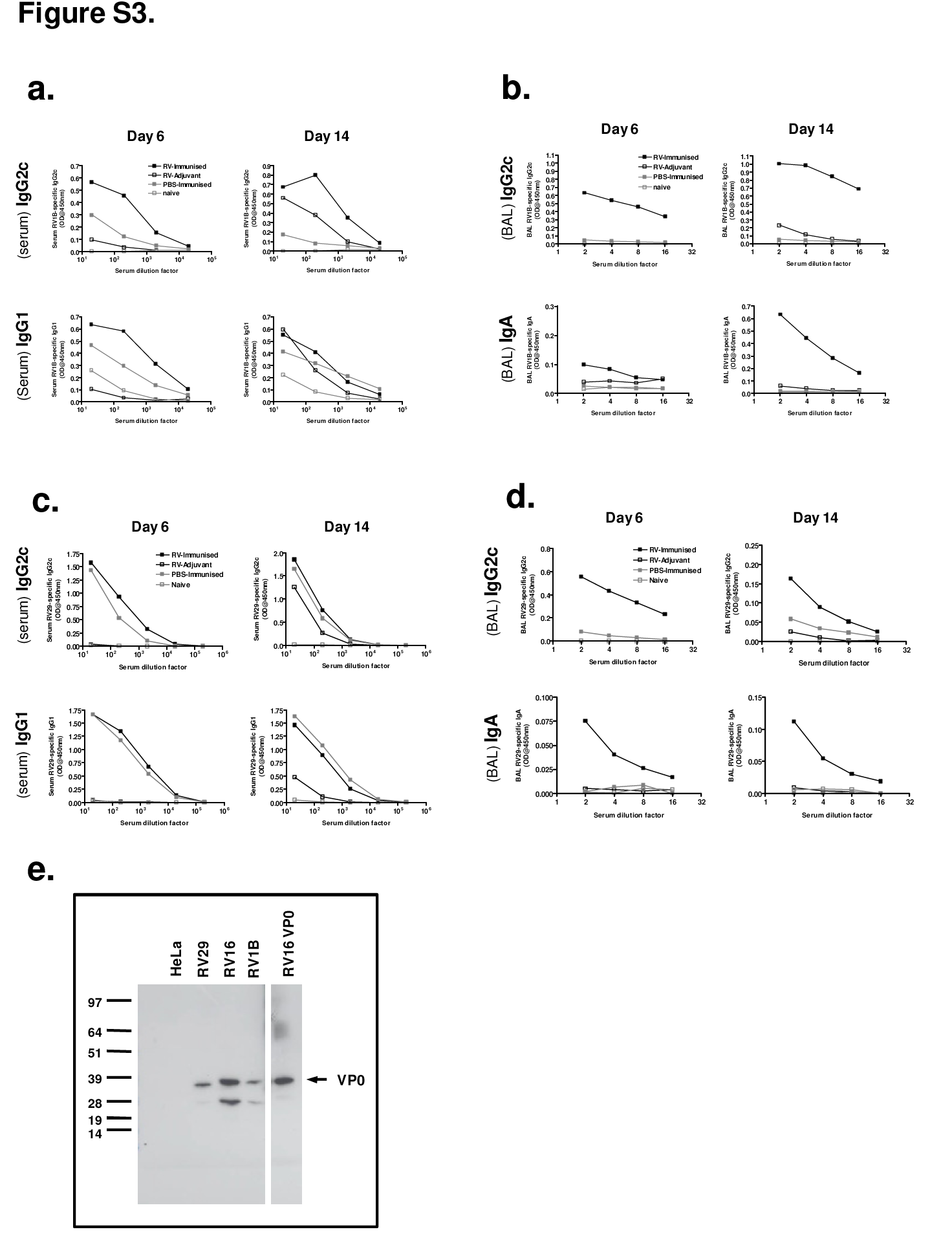

Supplement: Figure S3 — Serum and BAL antibody responses. (a–d) Mice were immunized subcutaneously with RV16 VP0 protein plus IFA/CpG, or with IFA/CpG adjuvant alone and infected intranasally with RV1B, RV29 or sham infected with PBS, as described. Sera and BAL were harvested at 6 and 14 days post-infection, pooled and assayed for IgG and IgA binding to virus inoculum preparations. (a) Serum and (b) BAL RV1B binding in RV1B-infected or PBS-challenged mouse sera. (c) Serum and (d) BAL RV29 binding in RV29-infected or PBS-challenged mouse sera. (e) Mice were immunized twice with RV16 VP0 protein plus IFA/CpG adjuvant subcutaneously and serum was harvested 6 weeks after immunization. Serum IgG binding to RV16 VP0 immunogen or to RV1B, RV29 and RV16 was assessed by Western blot. HeLa; virus culture cell lysate control. VP0; viral VP0 protein band estimated by molecular weight. (TIF) [file ppat.1003669.s003.tif]
